# Nanopore targeted sequencing-based diagnosis of central nervous system infections in HIV-infected patients

**DOI:** 10.1186/s12941-024-00682-7

**Published:** 2024-02-29

**Authors:** Xihong Yang, Shuilian Zhou, Ziwei Chang, Xiaotong Xi, Jiahui Li, Mengjiao Miao, Yaling Chen, Wei Chen, Hongying Zhang, Ran Ding, Zhiliang Hu

**Affiliations:** 1 Department of Infectious Disease, The Second Hospital of Nanjing, Nanjing University of Chinese Medicine, Nanjing, China; 2 State Key Laboratory of Neurology and Oncology Drug Development, Jiangsu Simcere Pharmaceutical Co.,Ltd, Jiangsu Simcere Diagnostics Co.,Ltd., Nanjing, China; 3 Nanjing Simcere Medical Laboratory Science Co., Ltd., Nanjing, China; 4 Center for Global Health, School of Public Health, Nanjing Medical University, Nanjing, China; 5 Clinical Research Center, The Second Hospital of Nanjing, Nanjing University of Chinese Medicine, Nanjing, China; 6 Nanjing Center for Disease Control and Prevention, Nanjing Medical University, Nanjing, China; 7 Inovention Center for Infectious Disease of Jiangsu Province, Nanjing, China

**Keywords:** Nanopore targeted sequencing, Central nervous system (CNS) infections, HIV, Cryptococcal meningitis

## Abstract

**Background:**

Early and accurate etiological diagnosis is very important for improving the prognosis of central nervous system (CNS) infections in human immunodeficiency virus (HIV)-infected patients. The goal is not easily achieved by conventional microbiological tests. We developed a nanopore targeted sequencing (NTS) platform and evaluated the diagnostic performance for CNS infections in HIV-infected patients, with special focus on cryptococcal meningitis (CM). We compared the CM diagnostic performance of NTS with conventional methods and cryptococcal polymerase chain reaction (PCR).

**Methods:**

This study included 57 hospitalized HIV-infected patients with suspected CNS infections from September 2018 to March 2022. The diagnosis established during hospitalization includes 27 cases of CM, 13 CNS tuberculosis, 5 toxoplasma encephalitis, 2 cytomegalovirus (CMV) encephalitis and 1 Varicella-zoster virus (VZV) encephalitis. The 2 cases of CMV encephalitis also have co-existing CM. Target-specific PCR amplification was used to enrich pathogen sequences before nanopore sequencing. NTS was performed on stored cerebrospinal fluid (CSF) samples and the results were compared with the diagnosis during hospitalization.

**Results:**

53 (93.0%) of the patients were male. The median CD4 cell count was 25.0 (IQR: 14.0–63.0) cells/uL. The sensitivities of CSF culture, India ink staining, cryptococcal PCR and NTS for CM were 70.4% (95%CI: 51.5 − 84.1%), 76.0% (95%CI: 56.6 − 88.5%), 77.8% (59.2 − 89.4%) and 85.2% (95%CI: 67.5 − 94.1%), respectively. All those methods had 100% specificity for CM. Our NTS platform could identify Cryptococcus at species level. Moreover, NTS was also able to identify all the 5 cases of toxoplasma encephalitis, 2 cases of CMV encephalitis and 1 VZV encephalitis. However, only 1 of 13 CNS tuberculosis cases was diagnosed by NTS, and so did Xpert MTB/RIF assay.

**Conclusions:**

NTS has a good diagnostic performance for CM in HIV-infected patients and may have the ability of simultaneously detecting other pathogens, including mixed infections. With continuing improving of the NTS platform, it may be a promising alterative microbiological test for assisting with the diagnosis of CNS infections.

**Supplementary Information:**

The online version contains supplementary material available at 10.1186/s12941-024-00682-7.

## Introduction

Central nervous system (CNS) infections contribute to substantial morbidity and mortality in Human immunodeficiency virus (HIV)-infected severely immunocompromised patients [1–3]. Early and accurate pathogen identification from cerebrospinal fluid (CSF) is very important for improving the prognosis of CNS infections. However, this goal is not easily achieved by using conventional microbiological tests, mainly involving morphological, serological, molecular, and culture methods. The CSF culture is time-consuming and many pathogens are not culturable by clinical labs. Polymerase chain reaction (PCR) assays are predominantly targeting very few pathogens and require a prior knowledge of the causative agents. It is not possible to test all possible pathogens using conventional microbiologic tests due to CSF volume restriction and commercial kits availability.

Recently, metagenomic next-generation sequencing (NGS), an agnostic, unbiased, and comprehensive method for pathogen identification, has attracted much attention in infectious field [4, 5]. The clinical utility of this strategy in etiological diagnosing of infectious diseases has been widely evaluated. In most scenarios, metagenomic NGS has similar to or even better sensitivity for pathogen identification compared with conventional microbiological methods [6–13]. However, metagenomic NGS predominantly using short-read sequencing platform also have limitations. Although metagenomic NGS can theoretically detect all the pathogens in a single run, the short-reading metagenomic sequencing platform is still relatively time-consuming with the overall turnover time of generally more than 24 h [14, 15]. Moreover, the sensitivity of metagenomic NGS is heavily influenced by background human genome material and contamination by environmental microorganisms usually complicates the clinical interpretating of the results [4].

Compared with short-reading NGS platform, long-reading NGS platform, for example Oxford Nanopore Technologies platform, has the advantages of portability, long read length, real-time analysis and short turn over time [5]. The overall sequencing process of Nanopore was optimized, with the detection cycle of 6 h, which met the clinical demand for rapid detection of pathogens in acute and critically ill patients to a greater extent [16, 17]. Recent studies have suggested that long-reading metagenomic nanopore sequencing may have similar performance as short-reading metagenomic NGS for diagnosing CNS or respiratory infections [18, 19]. Nevertheless, Nanopore sequencing is technically more difficult to detect pathogens with low nucleic acid load in the clinical samples, because there is no bridge PCR amplification process. Also, presence of massive host genome and contamination of environmental microorganisms are also great challenges impeding the clinical application of Nanopore sequencing [5].

Confronting the disadvantages of metagenomic NGS, we have developed a Nanopore targeted sequencing (NTS) platform where nucleic acid segments of common CNS pathogens in immunocompromised patients are enriched through pathogen-specific PCR before Nanopore sequencing. In the present study, we aimed to explore the diagnostic performance of NTS for CNS infections in HIV-infected patients with special focus on cryptococcal meningitis (CM). We also compared the CM diagnostic performance of NTS with conventional methods and cryptococcal PCR.

## Patients and methods

### Patients and study design

This study included 57 hospitalized HIV-infected patients who were admitted to the second hospital of Nanjing, China, from September 2018 to March 2022. The setting is a tertiary referral hospital and is a designated hospital that provides HIV care in Nanjing.

Since the study particularly focused on the diagnostic performance of NTS for cryptococcal meningitis in HIV-infected patients, HIV-infected patients tested positive for serum cryptococcal antigen (CrAg) assay with residuals CSF samples available (*n* = 32) during the aforementioned study period were included in this study. To investigate whether the NTS platform could simultaneously identified other CNS pathogens, we enrolled patients with the clinical diagnosis of CNS tuberculosis (*n* = 13) and toxoplasma encephalitis (*n* = 5) that had enough residual CSF samples. Other HIV patients (*n* = 7), deemed to be not having CM, CNS tuberculosis or toxoplasma encephalitis, were also included in this study.

Lumbar puncture was performed in 44 (77.2%) patients due to neurological symptoms suggested of CNS infections. For the 13 (22.8%) patients without obvious neurological symptoms, lumbar puncture was performed to screen for possible CNS involvement because the patients had positive serum CrAg assay, positive serum treponema pallidum hemagglutination test or disseminated tuberculosis. CSF samples were sent for conventional laboratory tests, and the remaining samples were stored at − 80 ℃ refrigerator for research purpose with informed consent obtained from the patients. The demographic, clinical and laboratory data of the patients were retrospectively collected from an electronic health record system. This study was approved by the ethics committee of the second hospital of Nanjing (reference number: 2020-LY-kt061).

### Procedure

In our routine clinical care, all CSF samples were sent to clinical laboratory for cell count, biochemistry and culture. The treating physicians decided which pathogen-specific mycobacterial tests should be performed based on the clinical condition of the patients. For patients suspected of CM, fresh CSF samples were sent for India ink staining, CrAg lateral flow assay (LFA, IMMY, Norman, USA), and cryptococcal culture. The reference CM cases should meet at least one of the following criteria: (1) positive CSF India ink staining; (2) positive CSF CrAg assay; or (3) positive CSF cryptococcal culture [20, 21].

**Fig. 1:**
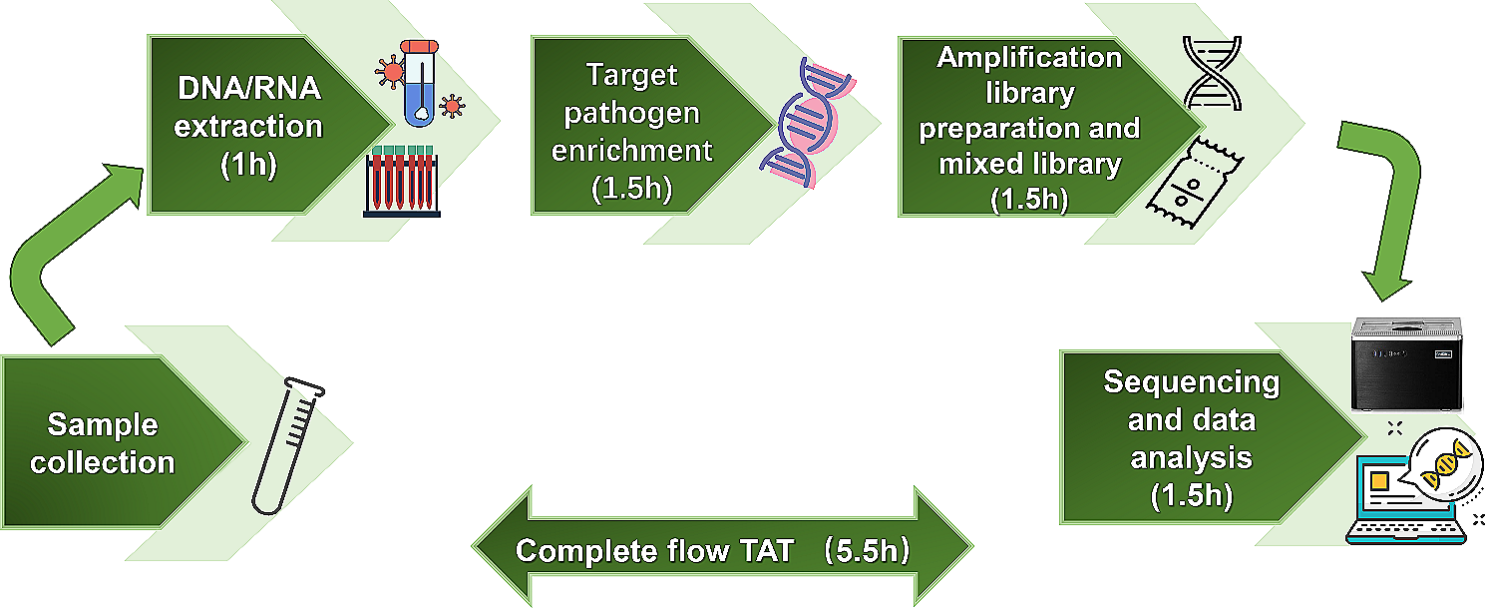
Procedure of nanopore targeted sequencing. The clinical application of the nanopore targeted sequencing (NTS) workflow, which consists of five parts. (1) Sample collection: cerebrospinal fluid of suspected meningitis patients was collected. (2) Nucleic acid extraction: cerebrospinal fluid sample DNA and RNA nucleic acid extraction. (3) Targeted pathogen enrichment: specific polymerase chain reaction (PCR) targeted enrichment of target pathogenic microorganisms. (4) Amplicon library preparation and mixed library: libraries were constructed for all samples using an SQK-PBK004 kit and library mixing was performed. (5) Sequencing and data analysis: library pools were loaded onto Gridion×5 sequencer for 1 h, and original data was analyzed

In the present study, nanopore targeted sequencing (Simceredx, Nanjing, China) was performed on stored CSF samples. Nucleic acid was extracted from 500 uL CSF samples using a Quick-DNA/RNA Viral Kit (D7021, Zymo Research) following the manufacturer’s instructions using 50 uL elution buffer. 30 uL nucleic acid was used for NTS. Before NTS, a preceding target-specific PCR amplification step was performed to enrich more than 30 common CNS pathogens (Supplemental Table 1) using target library kit (Jiangsu Simceredx Medical Equipment Co., Ltd.). The principles of designing PCR primers for the enrichment step included length of primer 18-21 bp, GC content of 40-60%, primer melting temperature of 50–70℃, preferable length of target fragment 1–2 kb, and covering conservative and variable intervals. Libraries were constructed for all samples using an SQK-PBK004 Kit (Oxford Nanopore Technologies). Library pools were then loaded onto an Gridion×5 (Oxford Nanopore Technologies) sequencer for 1 h (Fig. 1). Clean reads were obtained after removal of short reads (length less than 500 bp) and low-quality reads (mean q-score less than 8). Subsequently, reads for the host DNA were removed by aligning to the human reference genome (GRCh38) using minimap2 (Version 2.14-r883). The remaining reads were mapped using the Centrifuge software (version 1.0.4) for taxonomic classification. After the sequencing and analysis were completed, a Pubmed search was conducted to determine whether the detected species cause central nervous system infection. Before being applied to clinical samples, the designed NTS platformed had been evaluated for the limit of detection and analytical specificity in selected pathogens, including *Mycobacterium tuberculosis*, *Cryptococcus neoformans*, Human alphaherpesvirus 1, and Coxsackievirus A16. The results, provided in Supplemental Tables 2 and 3, suggests that this novel strategy was very sensitive and specific to identify pathogens.

With regarding to the evaluation of the performance of NTS on clinical samples, the positive results were verified by pathogen-specific qPCR. The detailed regents were provided in Supplemental Table 4. As for a positive detection of cryptococcal reads by NTS, it would be verified with a commercialized qPCR-based assay for cryptococcal DNA elements (Huayin Biotech, China). Clinical data and other test results were unavailable to those performing each NTS and cryptococcal PCR.

### Statistical analysis

Categorical variables were shown as frequencies and proportions. Continuous variables were expressed as medians and interquartile ranges (IQRs). Comparisons between different groups were performed using the chi-square test, Fisher’s exact test, or Mann–Whitney U test, when appropriate. Diagnostic performance (sensitivity, specificity, and positive and negative predictive values) of NTS for CM with the associated Wilson CIs were calculated. Sensitivities of different diagnostic methods were compared using McNemar’s test. All reported P values were 2-sided with the significance level of 0.05. R version 4.1.3.2 (R Foundation for Statistical Computing; www.r-project.org) was used for statistical analysis.

## Results

### Characteristics of the patients

Among 57 HIV-infected patients in the study, 53 (93.0%) were male and the median age was 33.0 (IQR: 29.0–46.0) years (Table 1). The median CD4 + T lymphocyte count was 25.0/ uL (IQR: 14.0–63.0), which was lower in patients with CM than in patients without it (*p* < 0.05). 47 (82.5%) patients had low CD4 + T cells count of < 100/ uL, which included all 27 patients with CM according to consensus definition. The majority of the patients were not on antiretroviral treatment (ART), and only 4 (7.4%, 4/54) patients achieved viral suppression (HIV RNA < 50 copies/mL). Serum CrAg assays were positive in 32 patients, of which 30 had semi-quantitative CrAg titers. Patients with CM (*n* = 25) had much higher serum CrAg titers than those without CM (*n* = 5) (*p* < 0.001). Of the 27 CM cases, 19 (70.4%) had a serum CrAg titer of ≥ 1:2560 and 12 (44.4%) were on anti-fungal therapy at the time of CSF collection, with a median duration of antifungal therapy of 4 (IQR:2–6) days. Other CNS infections of the participants were summarized in Table 1.

**Table 1:** Demographic and laboratory characteristics of the patients

Characteristics	Total (*n* = 57)	Cryptococcal meningitis		P value
		No (*n* = 30)	Yes (*n* = 27)	
Age	33.0[29.0,46.0]	31.0[29.0,46.8]	39.0[31.5,45.5]	0.263
Sex				
Male	53(93.0)	28(93.3)	25(92.6)	1
Female	4(7.0)	2(6.7)	2(7.4)	
HBV coinfection	5(8.8)	2(6.7)	3(11.1)	0.66
HCV coinfection	1(1.8)	1(3.3)	0(0.0)	1
TP	18(31.6)	11(36.7)	7(25.9)	0.558
CD4 + T cells count (/uL)	25.0[14.0,63.0]	55.0[19.2,139.8]	17.0[11.0,31.5]	0.001
HIV RNA(lg copies/mL)	4.9[4.2,5.3]	4.8[1.9,5.0]	5.0[4.7,5.4]	0.116
< 50 copies /mL	4(7.4)	3(10.3)	1(4.0)	0.615
Missing data	3(5.3)	1(3.3)	2(7.4)	0.599
ART status				
Not on ART	38(66.7)	16(53.3)	22(81.5)	0.03
ART ≤ 30 days	6(10.5)	3(10.0)	3(11.1)	
ART > 30 days	13(22.8)	11(36.7)	2(7.4)	
Neurological symptoms	44(77.2)	19(63.3)	25(92.6)	0.011
CSF analysis				
CSF WBC (/uL)	6.0[1.0,22.0]	5.5[1.2,21.5]	7.0[2.0,24.5]	0.399
CSF protein (mg/L)	486.0[312.2,689.0]	448.6[332.8,675.5]	503.0[272.6,808.5]	0.867
CSF LDH (U/L)	29.0[20.0,49.0]	22.5[19.2,34.0]	34.0[24.5,70.5]	0.018
CSF glucose (mmol/L)	2.6[2.2,3.1]	2.8[2.5,3.5]	2.3[1.5,3.0]	0.012
CSF Cl (mmol/L)	122.0[117.0,126.0]	122.5[119.0,125.8]	122.0[117.0,125.5]	0.625
CSF ADA (U/L)	2.0[1.0,4.0]	1.3[0.2,3.0]	3.0[1.5,5.2]	0.025
Other CNS infections^1^				
CNS tuberculosis	13(22.8)	13(43.3)	0(0.0)	
Toxoplasma encephalitis	5(8.8)	5(16.7)	0(0.0)	
CMV encephalitis	2(3.5)	0(0.0)	2(7.4)	
VZV encephalitis	1(1.8)	1(3.3)	0(0.0)	
Neurosyphilis	1(1.8)	1(3.3)	0(0)	
No CNS infection^2^	11(19.3)	11(36.7)	0(0.0)	

Data were described as median [IQR] or no. (%). P values were derived from chi-square test, Fisher’s exact test or Mann–Whitney U test, when appropriate

Abbreviations: HBV, Hepatitis B virus; HCV, Hepatitis C virus; TP, Treponema Pallidum; HIV, human immunodeficiency virus; ART, antiretroviral therapy; CSF, cerebrospinal fluid; LDH, lactic dehydrogenase; ADA, adenosine deaminase; CNS, central nervous system; CMV, cytomegalovirus; VZV, Varicella-zoster virus

1. The patient with VZV encephalitis was also clinically diagnosed with CNS tuberculosis

2. Those 11 patients received lumbar punctures due to positive serum cryptococcal antigen assay or positive serum treponema pallidum hemagglutination test

### Diagnostic performance

**Fig. 2:**
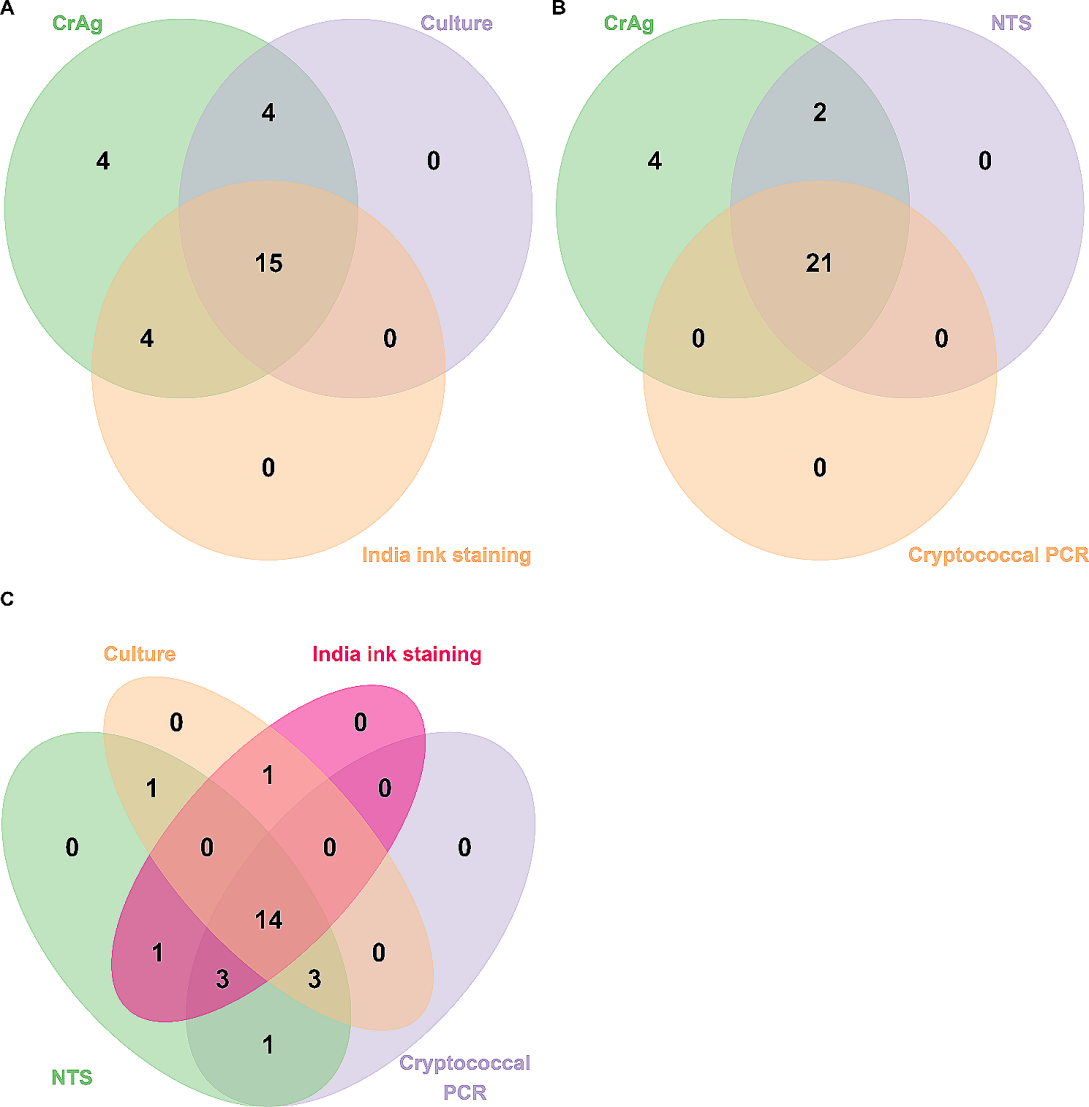
Venn diagrams of positive results of different methods. Positive results of different methods in 27 cryptococcal meningitis cases, including cryptococcal antigen (CrAg), culture, India ink staining, nanopore targeted sequencing (NTS), polymerase chain reaction (PCR). (**A**) CrAg, culture, India ink staining. (**B**) CrAg, NTS, PCR. (C) culture, India ink staining, NTS, PCR.

**Fig. 3:**
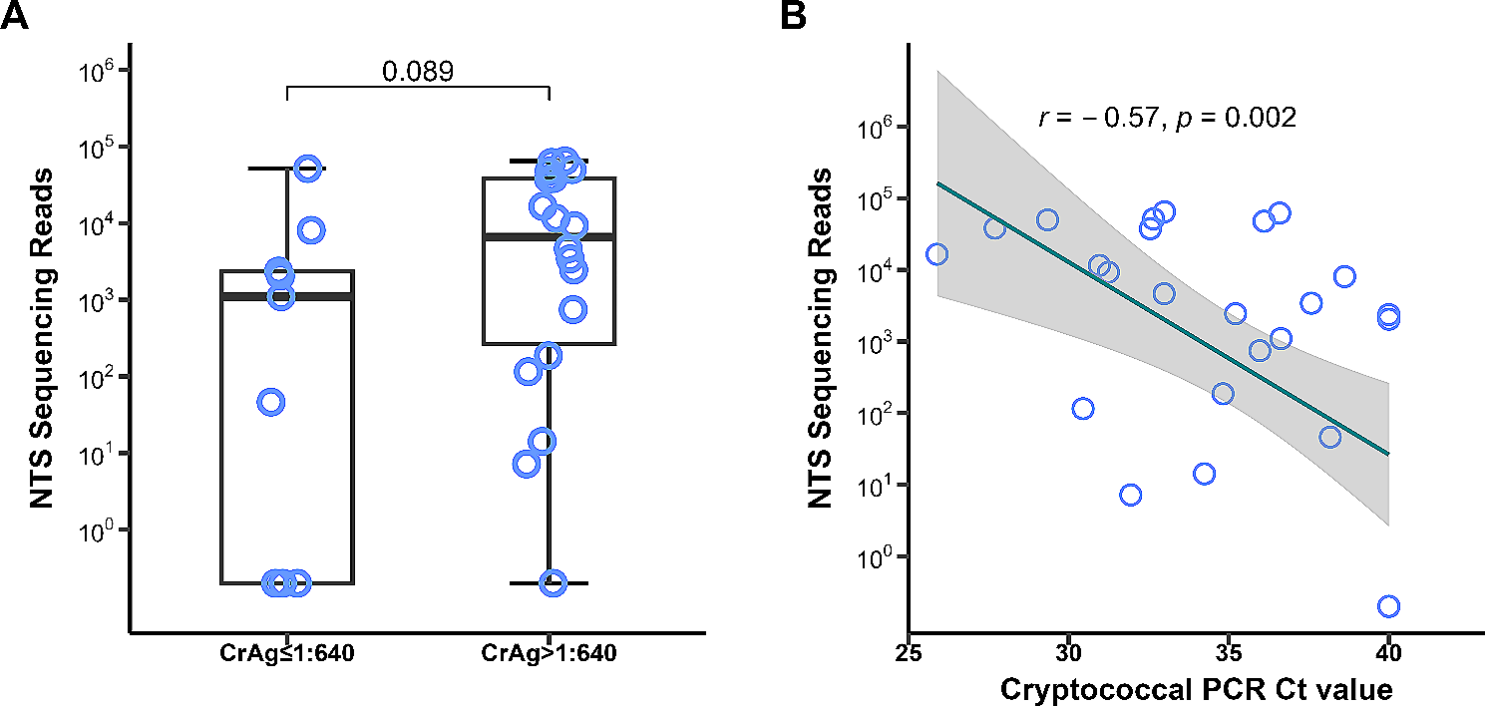
Associations of cryptococcal sequencing reads with CrAg and PCR. (**A**) The comparation of nanopore targeted sequencing (NTS) sequencing reads between groups of cerebrospinal fluid (CSF) cryptococcal antigen (CrAg) titer ≤1:640 and CSF CrAg titer > 1:640 of 27 patients with cryptococcal meningitis, and NTS sequencing reads of each case. (**B**) The relationship between NTS sequencing reads and cryptococcal PCR cycle threshold (Ct) value of 28 patients. Of 7 cases with negative PCR results, the Ct value was considered as maximum thermal cycles of 40. In both A and B, when NTS sequencing reads was 0, it was adjusted to 0.2 for the purpose of analysis

Of the three widely used traditional microbiological tests, CSF CrAg was positive in all the 27 references CM cases. CSF culture and CSF India ink staining failed to detect 8 (29.6%) and 6 (24.0%, 6/25; India ink staining was not performed in 2 culture positive cases) CM cases, respectively (Fig. 2A). NTS was able to detect 23 (85.2%) CM cases, and 2 of those 23 cases could not be confirmed by cryptococcal PCR (Fig. 2B). When all the diagnostic tests were considered, 14 CM cases were tested positive by all of those tests (Fig. 2C). Consequently, the sensitivities of CSF CrAg, CSF culture, CSF India ink staining, CSF cryptococcal PCR and CSF NTS for diagnosing were 100% (95%CI: 87.5 − 100%), 70.4% (95%CI: 51.5 − 84.1%), 76.0% (95%CI: 56.6 − 88.5%), 77.8% (59.2 − 89.4%) and 85.2% (95%CI: 67.5 − 94.1%), respectively (Table 2). Patients with higher CSF CrAg titers seemed to have higher cryptococcal sequencing reads reported by CSF NTS (*p* = 0.089; Fig. 3), and there was moderated correlation between cryptococcal PCR Ct value and NTS cryptococcal sequencing reads (Spearman’s *r* = 0.57; *p* = 0.002; Fig. 3). The pairwise comparison of the sensitivities between different tests were shown in Supplemental Table 5. There was no statistical significance of the sensitivity between CSF CrAg and CSF NTS for diagnosing CM among HIV-infected patients (*p* = 0.134; Supplemental Table 5). All the tests had high specificity (100%) that a positive result could confirm a CM case (Table 2).

**Table 2:** Diagnostic performance for cryptococcal meningitis

Test	Sensitivity (95% CI; n/N)	Specificity (95% CI; n/N)	PPV (95% CI; n/N)	NPV (95% CI; n/N)
CrAg	100.0 (87.5–100.0; 27 / 27)	100.0 (86.7–100.0; 25 / 25)	100.0 (87.5–100.0; 27 / 27)	100.0 (86.7–100.0; 25 / 25)
Culture	70.4 (51.5–84.1; 19 / 27)	100.0 (88.6–100.0; 30 / 30)	100.0 (83.2–100.0; 19 / 19)	78.9 (63.7–88.9; 30 / 38)
India ink staining	76.0 (56.6–88.5; 19 / 25)	100.0 (84.5–100.0; 21 / 21)	100.0 (83.2–100.0; 19 / 19)	77.8 (59.2–89.4; 21 / 27)
Cryptococcal PCR	77.8 (59.2–89.4; 21 / 27)	100.0 (20.7–100.0; 1 / 1)	100.0 (84.5–100.0; 21 / 21)	14.3 (2.6–51.3; 1 /7)
NTS	85.2 (67.5–94.1; 23 / 27)	100.0 (88.6–100.0; 30 / 30)	100.0 (85.7–100.0; 23 / 23)	88.2 (73.4–95.3; 30 / 34)

The diagnosis of cryptococcal meningitis is based on a combination of all microbiological examination results. CSF CrAg, CSF Culture, CSF India ink staining, CSF Cryptococcal PCR and CSF NTS were done in 52, 57, 46, 28 and 57 patients respectively

Abbreviations: NTS, Nanopore targeted sequencing; CI, Confidence interval; PPV, Positive predictive value; NPV, Negative predictive value

### Other pathogens reported by NTS

**Fig. 4:**
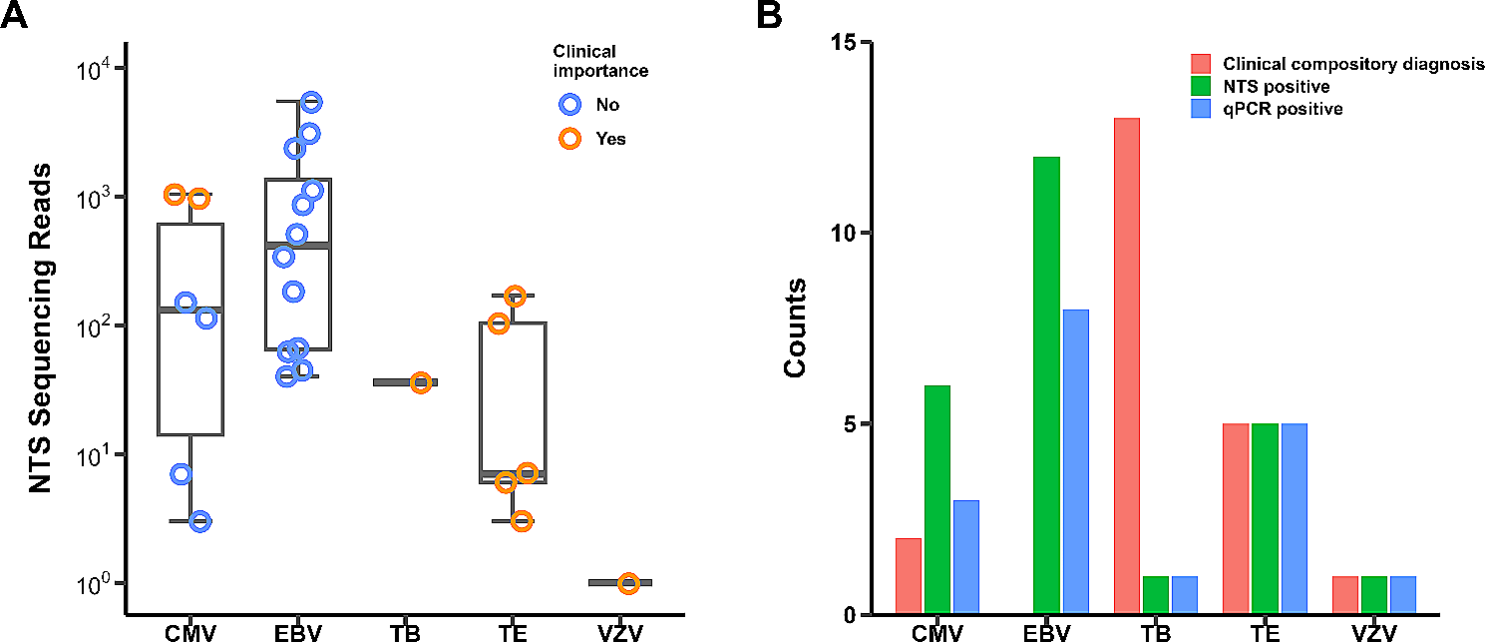
Other pathogens reported by nanopore targeted sequencing. (**A**) Pathogens except cryptococcus that detected by nanopore targeted sequencing (NTS), and the NTS sequencing reads of each case. Grouped by whether the detection was clinically important. (**B**) Comparations of clinically composite diagnosed cases, positive NTS and polymerase chain reaction (PCR) cases of each pathogen

Among 27 patients diagnosed with CM, *Cryptococcus neoformans* and *Cryptococcus gattii* were reported in 22 and 1 patients respectively. Aside from cryptococcal species, NTS detected sequences of cytomegalovirus (CMV) in 6 patients, *Epstein-barr* virus (EBV) in 12 patients, *Mycobacterium tuberculosis* (MTB) in 1 patient, *Toxoplasma gondii* in 5 patients and V*aricella-zoster* virus (VZV) in 1 patient (Fig. 4A). The detections of those microorganisms were compared with specific real-time quantitative PCR and clinical composite diagnosis (summarized in Table 1) at the time of discharge (Fig. 4B). Of the 6 positive detections of CMV by NTS, 3 was positive for CMV-specific PCR. However, only 2 patients were considered as CMV encephalitis by clinical composite diagnosis and treated with anti-CMV treatment during hospitalization. Importantly, those two cases of CMV encephalitis were also diagnosed with CM (Table 1). Of the other 4 patients with positive CMV detections by NTS however not considered active CMV encephalitis, 1 was diagnosed with neurosyphilis (positive CSF rapid plasma reagin and Treponema pallidum particle agglutination tests) and 1 had history treated CMV encephalitis three months ago. The other 2 patients did not have obvious neurological symptoms and none of them were diagnosed as CMV encephalitis during hospitalization.

Of the 12 positive detections of EBV by NTS, 8 was positive for EBV-specific PCR. None of those 12 positive detections were considered clinical important because patients were clinically stable at the time of discharge and EBV-related diseases were not identified. There were 5 cases of TE by clinical composite diagnosis. For those 5 patients, serum anti-toxoplasma IgG antibody were positive and *Toxoplasma gondii* were detected by CSF metagenomic NGS. In our study, NTS were able to identify all those cases (Fig. 4B).

At the time of discharge, 13 patients were diagnosed with CNS tuberculosis (Table 1), of which only 2 were microbiological confirmed (positive CSF mycobacterial culture). The diagnosis of CNS tuberculosis in the remaining 11 patients were extensively investigated. Among those 11 patients, CNS tuberculosis were diagnosed in 10 patients based on the presence of abnormal magnetic resonance imaging (MRI) changes consistent with CNS tuberculosis with or without neurological symptoms, the identification of MTB from other samples, the favorable response to anti-tuberculosis treatment and the exclusion of other CNS infections. Clinical diagnosis of CNS tuberculosis was very difficult in 1 patient with pulmonary tuberculosis (positive sputum Xpert MTB/RIF assay) and shingles. The neurological symptoms resolved with anti-tuberculosis and anti-VZV treatment, therefore the patient may be clinically diagnosed as CNS tuberculosis and/or VZV encephalitis. In our study, only 1 of the 13 CNS tuberculosis cases could be identified by NTS (Fig. 4B), and so did Xpert MTB/RIF assay.

## Discussion

Recently, PCR amplification of conserved regions (16 S rRNA gene of the bacteria and internal transcribed spacer region of fungi) has been applied for enrichment of pathogen gene sequences in NTS process [22–24]. This strategy has been found to facilitate early diagnosing of bacterial or fungal infections and could guide optimization of antimicrobial therapy [22–24]. The merit of this strategy is that only a few PCR primers are required for bacterial or fungal sequences enrichment. Nevertheless, the afore-mentioned strategy is not suited for viral infections, because virus does not contain ribosome. Therefore, to enrich viral sequences, specific PCR primers are generally needed for each type of virus. In our study, we designed a NTS platform where each type of virus would be amplified through PCR with pathogen-specific primers. This platform also enriches DNA sequences of mycobacterium, aspergillus, cryptococci and *Toxoplasma gondii* through pathogen-specific PCR amplification (Supplemental Table 1). Consequently, the NTS panel covered common opportunistic CNS infections in HIV-infected patients as described in other and our studies [2, 7, 12, 25].

The present study was designed to preliminarily evaluate the performance of NTS for diagnosis of CNS infections in HIV-infected patients. Since CM is a common and well recognized opportunistic CNS infection in HIV-infected patients [2, 3], the primary goal of this study was to explore the diagnostic performance of NTS for CM. It is well recognized that CSF CrAg LFA is the most sensitive test for diagnosing CM, with sensitivity and specificity of almost 100% [26, 27]. What is not unexpected that NTS missed a few cases of CM which could be identified by CrAg LFA; however, it detected more CM cases than culture, India ink staining or cryptococcal PCR (Fig. 2; Table 2). The sensitivity and specificity of NTS for CM in HIV-infected patients were 85.2% (95%CI: 67.5 − 94.1%) and 100% (95% CI: 88.6 − 100.0%), respectively. Moreover, our NTS identified cryptococcus at species level and had the capability to simultaneously detect other pathogens. Although data on NTS’s role in diagnosing CM are limited, several studies have highlighted the effectiveness of metagenomic next-generation sequencing for CM diagnosis, with sensitivities ranging from 75 to 93.5% [28–30]. These studies, along with our own, indicate that molecular-based methods, despite their high diagnostic accuracy for CM, are not infallible. Factors such as the thick cryptococcal capsule, which may hinder DNA extraction, and low CSF cryptococcal DNA burden in some cases, could lead to false-negative results in cryptococcal molecular tests. Finally, the NTS panel used in this study was able to correctly identify all the 5 cases of toxoplasma encephalitis, 2 case of CMV encephalitis and 1 VZV encephalitis (Fig. 4B). Together, those results suggests that NTS may be a promising alternative for diagnosing CNS infections in HIV-infected patients.

CMV and EBV could establish life-long persistence and may be detected in peripheral blood mononuclear cells of the healthy blood donors [31–33]. It is not uncommon that those viruses are present in the CSF of HIV-infected patients and metagenomic NGS is likely to be more sensitive than specific PCR methods to identify those viruses [7, 12, 25]. However, identification of CMV and EBV is not always suggestive of active associated disease [7, 12, 25]. In our study, NTS also reported more CMV and EBV positive cases than specific PCR methods, although only two positive CMV detections were considered as clinically important. Taken together, our NTS platform is very sensitive for detecting CMV or EBV in the CSF samples. Nevertheless, caution is needed in interpreting the positive detections of CMV or EBV by NTS platform because most of the positive detections are not associated with active diseases.

Unlike the diagnosing of CM, the diagnosing of CNS tuberculosis remains challenging. Currently, Xpert MTB/RIF assay is the preferred initial test for extrapulmonary tuberculosis [34]. Previous studies have demonstrated that the sensitivity of CSF Xpert MTB/RIF assay for tuberculosis meningitis in HIV-infected patients ranges from 43 to 76.9% [35, 36]. The present study included 13 HIV-associated CNS tuberculosis, but only 1 CSF sample was tested positive for MTB by using NTS. Technical limitations are unlikely to be the sole cause of our NTS platform’s diagnostic shortcomings, as evidenced by the Xpert MTB/RIF assay also confirming only one case of CNS tuberculosis in this study. Perhaps, the CNS tuberculosis cases included in this study were in the early stage of CNS involvement with low mycobacterial burden in the CSF.

There are some limitations in our study. Since the inclusion of patients were largely influenced by the availability of the stored samples, the selection bias should not be overlooked. We used pathogen-specific PCR to enrich sequences of the pathogens before Nanopore sequencing, however, we were not able to evaluate the diagnostic performance of NTS for each CNS pathogen. Moreover, although pathogen-specific PCR primers are very suited for enrichment of viral sequences during NTS process, the number of pathogens that could be investigated by NTS is relatively small. At this point, our NTS panel could not be used for common bacterial meningitis. Future optimization of this NTS platform to facilitate diagnosing of bacterial meningitis may incorporate PCR amplification of conserved regions of bacterial 16 S rRNA before nanopore sequencing, as has been applied in previous studies [22, 24]. Alternatively, pathogen-specific PCR targeting the common bacteria involving in CNS infection could also be designed for nucleic acid enrichment. Nevertheless, which enrichment method is more suited for CNS infection has yet to be explored in future studies.

## Conclusion

In summary, we have developed a NTS platform where enrichment of pathogen sequences is achieved through pathogen-specific PCR amplification. This NTS platform is able to detect common CNS pathogens in the CSF samples of HIV-infected patients. Since NTS requires less data than metagenomic sequencing, it would greatly save the cost of the test. With continuing improving of the NTS platform, it may be an important alterative microbiological test for assisting with the diagnosis of CNS infections.

### Electronic supplementary material

Below is the link to the electronic supplementary material.

Supplementary Material 1

Supplementary Material 2

Supplementary Material 3

Supplementary Material 4

Supplementary Material 5

## Data Availability

The datasets used and/or analyzed during the current study are available from the corresponding author on reasonable request.

## Abbreviations

CNS: central nervous system
HIV: human immunodeficiency virus
CSF: cerebrospinal fluid
PCR: polymerase chain reaction
NGS: next-generation sequencing
NTS: nanopore targeted sequencing
CM: cryptococcal meningitis
CrAg: cryptococcal antigen
LFA: lateral flow assay
IQR: interquartile ranges
ART: antiretroviral treatment
CMV: cytomegalovirus
EBV: *Epstein-barr* virus
MTB: *Mycobacterium tuberculosis*
VZV: varicella-zoster virus
MRI: magnetic resonance imaging
