# Supplementary material for: Nanopore targeted sequencing-based diagnosis of central nervous system infections in HIV-infected patients
Source: Ann Clin Microbiol Antimicrob. 2024 Feb 29;23:22. doi: 10.1186/s12941-024-00682-7 (PMC10905896; doi:10.1186/s12941-024-00682-7)
Supplement: Supplementary file 1 — Supplementary Material 1 [file 12941_2024_682_MOESM1_ESM.docx]

**Supplemental table 1 List of pathogens and primers enriched by specific PCRs before nanopore targeted sequencing**

| Serial number | Species | Classify | Primer(5’-3’) |
| --- | --- | --- | --- |
| 1 | *Mycobacterium tuberculosis* | Bacteria | F:AGCAGGCATTGTTACCACAC |
|  |  |  | R:CCTCCTCGATGAACCACCT |
| 2 | *Mycobacterium* | Bacteria | F:TGCAAAGAYAAGGACATGACST |
|  |  |  | R:TGCTGVGTRATCATCGAGTACG |
| 3 | *Aspergillus flavus/ Aspergillus fumigatus/ Aspergillus niger* | Fungi | F:AGAAGGTYTGCGGTCTGATYG |
|  |  |  | R:ACGRGGAATCCAGGCGTTGTC |
| 4 | *Aspergillus flavus* | Fungi | F:CACCTGTGACGTGAGCCTA |
|  |  |  | R:ACCTGCGAATCCCACATACC |
| 5 | *Aspergillus fumigatus* | Fungi | F:ACAGGATTGGCTACAACAGG |
|  |  |  | R:CCTTCCATTTGAGGTGATGC |
| 6 | *Cryptococcus gattii* | Fungi | F:CGCCCTTCCTCTCCATACAT |
|  |  |  | R:GGTGTCCACAATCGTACCC |
| 7 | *Cryptococcus neoformans* | Fungi | F:CCCACCCTTTCATGGCTAC |
|  |  |  | R:GGGTTATCCGTCGTGATCCT |
| 8 | Epstein–Barr virus | DNA-virus | F:CCGTGAGATGGATCAGGCTC |
|  |  |  | R:CAGCAACAACCTGTGCAGAC |
| 9 | Cytomegalovirus | DNA-virus | F:CCTCCACCCACTACAACACAC |
|  |  |  | R:GTGATGTCCCTCGCAGAACCT |
| 10 | Human mastadenovirus | DNA-virus | F:GTRACRAAVARRCTGTCNGTGTCYCCGTA |
|  |  |  | R:CTACTTYCAYCAYATCAAYRSCCAHTC |
| 11 | Human alphaherpesvirus-1/2 | DNA-virus | F:GGTTGGAATTGRTTRCGAAAGTT |
|  |  |  | R:CTCCCCSCYGACATTACGTWCACG |
| 12 | Varicella-zoster virus | DNA-virus | F:GGCATTGGTCCAGTTACCTC |
|  |  |  | R:ACACCGTCTGCATGATTGAC |
| 13 | Human betaherpesvirus 6B | DNA-virus | F:TTTCGGGTAGCGTTCGTCTT |
|  |  |  | R:GGGCGGAGAAATGTAGAGAG |
| 14 | Human herpesvirus 7 | DNA-virus | F:TTCGGCTACTTGAGAAAGCG |
|  |  |  | R:TCAGCAGTGTACGGTAAACC |
| 15 | Human polyomavirus 1 (BK virus) | DNA-virus | F:CAGCTCCTGTTCCCTTCAC |
|  |  |  | R:ACCCTGTACTGCAAGGAATG |
| 16 | Human polyomavirus 2 (JC virus) | DNA-virus | F:CCCAAATGTGCAATCTGGTG |
|  |  |  | R:CTGGTGCAGAGTCAAGGGAT |
| 17 | Merkel cell polyomavirus | DNA-virus | F:GATCTCGCCTCAAACCTCAC |
|  |  |  | R:TGCCCTAATGTTGCCTCAGT |
| 18 | Toxoplasma gondii | Parasite | F:GAGCCACAGAAGGGACAGAAG |
|  |  |  | R:TCTGGATTCCTCTCCTACGCC |
| 19 | Japanese encephalitis virus | RNA-virus | F:TGGACCCGAGACAAAGGAAT |
|  |  |  | R:GCGGTCTTCCTTCACACTAC |
| 20 | Dengue virus | RNA-virus | F:AGTCGGGATGGTGAGCATTT |
|  |  |  | R:GTCTGCGTAGTTGATGCCTT |
| 21 | Zika virus | RNA-virus | F:GGCCGAGGTGAGATCCTAT |
|  |  |  | R:GCCTCCTTTCCCTTAACAGC |
| 22 | Chikungunya virus | RNA-virus | F:CATAGCCGCACACTTTAAGC |
|  |  |  | R:TCCCACCGTCAGAGTTTCC |
| 23 | Tick-borne encephalitis virus | RNA-virus | F:CCAGACCAAGATCCAACAGC |
|  |  |  | R:TGACCACCTGTTCTGCTCAT |
| 24 | West Nile virus | RNA-virus | F:GACGCATCGGTAGGAATCC |
|  |  |  | R:CCTCCAGTGACATTGCCTTC |
| 25 | Nipah henipavirus | RNA-virus | F:GGTTGCCTCCTTCATGCTAC |
|  |  |  | R:GGAGTCGTTGAGCCTCTTTG |
| 26 | Rabies lyssavirus | RNA-virus | F:CCCGATACTCCGATGACTTG |
|  |  |  | R:GGACAACCCTTCAGAGACAG |
| 27 | Enterovirus 71 | RNA-virus | F:TGCTGGGCACACATGTTATC |
|  |  |  | R:GGTGCGATTGGTATCTAGCG |
| 28 | Human enterovirus A | RNA-virus | F:CGATGTTTGGCAACGTGTCTT |
|  |  |  | R:GATCTCACCCTGTTCACTGC |
| 29 | Human enterovirus B | RNA-virus | F:GCCGTGGCTATGATGAAGAG |
|  |  |  | R:GTGAGTGTGGTAGGGTCAAG |
| 30 | Human enterovirus C | RNA-virus | F:CACCACGAACAAGCACTTCT |
|  |  |  | R:AGTTGCCATCATGCTACCAC |
| 31 | Human enterovirus D | RNA-virus | F:GGAGGAGTCTTTGTAGGG |
|  |  |  | R:GTGGAGGTCCCTGAAACAG |
| 32 | Coxsackievirus A16 | RNA-virus | F:CCCGTGTAGATCAGGTCGAT |
|  |  |  | R:AGCGGTTCACTTGGCTATTC |
| 33 | Severe fever with thrombocytopenia syndrome virus | RNA-virus | F:TGAGGGTAGTTGCATGTTCC |
|  |  |  | R:ACTCACGCCCTTGAAGAC |

R = A/G, Y = C/T, M = A/C, S = C/G, W = A/T, B = C/G/T, D = A/G/T, V=A/C/G, H=A/C/T, N=A/G/C/T
