# Supplementary material for: Nanopore targeted sequencing-based diagnosis of central nervous system infections in HIV-infected patients
Source: Ann Clin Microbiol Antimicrob. 2024 Feb 29;23:22. doi: 10.1186/s12941-024-00682-7 (PMC10905896; doi:10.1186/s12941-024-00682-7)
Supplement: Supplementary file 2 — Supplementary Material 2 [file 12941_2024_682_MOESM2_ESM.docx]

**Supplemental table 2 The limit of detection of different species.**

| **Category** | **Species** | **LoD** |
| --- | --- | --- |
| Bacteria | *Mycobacterium tuberculosis* | 150 CFU/mL |
| Fungi | *Cryptococcus neoformans* | 172 CFU/mL |
| DNA viruses | Human alphaherpesvirus 1 | 52 Copies/mL |
| RNA viruses | Coxsackievirus A16 | 49 Copies/mL |

To evaluate the limit of detection (LoD) for this assay, we spiked with serial tenfold dilutions (10–10^4^ CFU/mL or 10-10^4^ Copies/mL) of *Mycobacterium tuberculosis*, *Cryptococcus neoformans*, Human alphaherpesvirus 1, and Coxsackievirus A16 using uninfected normal CSF samples, which was negative of these spiked pathogens that confirmed by quantitative polymerase chain reaction (qPCR). Each concentration of the mixed microorganisms was tested in quintuplicate. LoD was defined as the lowest concentration at which spiked-in pathogens were detected ≥4 mock samples.
