# Supplementary material for: Nanopore targeted sequencing-based diagnosis of central nervous system infections in HIV-infected patients
Source: Ann Clin Microbiol Antimicrob. 2024 Feb 29;23:22. doi: 10.1186/s12941-024-00682-7 (PMC10905896; doi:10.1186/s12941-024-00682-7)
Supplement: Supplementary file 3 — Supplementary Material 3 [file 12941_2024_682_MOESM3_ESM.docx]

**Supplemental table 3 Specificity of representative primers**

|  | Reads | | | | | | | | | |
| --- | --- | --- | --- | --- | --- | --- | --- | --- | --- | --- |
|  | *Mycobacterium tuberculosis* | *Cryptococcus neoformans* | Human alphaherpesvirus 1 | Coxsackievirus A16 | *Pseudomonas aeruginosa* | *Staphylococcus aureus* | *Mycoplasma* | *Candida albicans* | Influenza A | Human Coronavirus NL63 |
| 1 | 298752 | NA | NA | NA | ND | ND | ND | ND | ND | ND |
| 2 | NA | 148181 | NA | NA | ND | ND | ND | ND | ND | ND |
| 3 | NA | NA | 17129 | NA | ND | ND | ND | ND | ND | ND |
| 4 | NA | NA | NA | 58174 | ND | ND | ND | ND | ND | ND |

NA: not available, no pathogens were incorporated; ND: not detected.

To evaluate specificity of primers, we selected Human alphaherpesvirus 1, Coxsackievirus A16, *Mycobacterium tuberculosis* and *Cryptococcus neoformans* as reference products. *Pseudomonas aeruginosa*, *Staphylococcus aureus*, *Mycoplasma*, *Candida albicans*, Influenza A virus and Human Coronavirus NL63 interference substances were added to the four reference products, and nucleic acids were extracted, respectively. The amplified libraries of each species are assigned a unique library barcode label, and the library quality hybrid libraries are sequenced on the same chip. The specificity of primers was evaluated through comparative analysis of target species reads in the experiment.
