# Supplementary material for: Nanopore targeted sequencing-based diagnosis of central nervous system infections in HIV-infected patients
Source: Ann Clin Microbiol Antimicrob. 2024 Feb 29;23:22. doi: 10.1186/s12941-024-00682-7 (PMC10905896; doi:10.1186/s12941-024-00682-7)
Supplement: Supplementary file 4 — Supplementary Material 4 [file 12941_2024_682_MOESM4_ESM.docx]

**Supplemental table 4 The methods of qPCR testing**

| **Species** | **Category** | **Manufacturer/Primer** | | **Probe** |
| --- | --- | --- | --- | --- |
| *Mycobacterium tuberculosis* | Bacteria | Zeesan, Zeesan Biotech Co., Ltd., Xiamen, China | | / |
| *Cryptococcus* | Fungi | Huayin, Shenzhen Huayin Biotech Co., Ltd., Shenzhen, China | | / |
| *Epstein–Barr virus* | DNA viruses | Sansure, Changsha Sansure Biotech Co., Ltd., Changsha, China | | / |
| *Cytomegalovirus* | DNA viruses | Sansure, Changsha Sansure Biotech Co., Ltd., Changsha, China | | / |
| *Varicella-zoster virus* | DNA viruses | Sansure, Changsha Sansure Biotech Co., Ltd., Changsha, China | | / |
| *Toxoplasma gondii* | Parasite | F: AGGAGAGATATCAGGACTGTAG | R: GCGTCGTCTCGTCTAGATCG | FAM-CCGGCTTGGCTGCTTTTCCTG-TAMRA |
