# Supplementary material for: Nanopore targeted sequencing-based diagnosis of central nervous system infections in HIV-infected patients
Source: Ann Clin Microbiol Antimicrob. 2024 Feb 29;23:22. doi: 10.1186/s12941-024-00682-7 (PMC10905896; doi:10.1186/s12941-024-00682-7)
Supplement: Supplementary file 5 — Supplementary Material 5 [file 12941_2024_682_MOESM5_ESM.docx]

| group1 | group2 | p value | adjusted p value |
| --- | --- | --- | --- |
| CrAg | culture | 0.013 | 0.133 |
| CrAg | India ink staining | 0.041 | 0.412 |
| CrAg | NTS | 0.134 | 1 |
| CrAg | PCR | 0.041 | 0.412 |
| culture | India ink staining | 0.683 | 1 |
| culture | NTS | 0.221 | 1 |
| culture | PCR | 0.683 | 1 |
| India ink staining | NTS | 0.617 | 1 |
| India ink staining | PCR | 1 | 1 |
| NTS | PCR | 0.480 | 1 |

**Supplemental table 5 Pairwise comparison of the sensitivities between different tests**

Abbreviations: CrAg, cryptococcal antigen; NTS, Nanopore targeting sequencing.
